# Recombinant AAV-Mediated *BEST1* Transfer to the Retinal Pigment Epithelium: Analysis of Serotype-Dependent Retinal Effects

**DOI:** 10.1371/journal.pone.0075666

**Published:** 2013-10-15

**Authors:** Karina E. Guziewicz, Barbara Zangerl, András M. Komáromy, Simone Iwabe, Vincent A. Chiodo, Sanford L. Boye, William W. Hauswirth, William A. Beltran, Gustavo D. Aguirre

**Affiliations:** 1 Section of Ophthalmology, School of Veterinary Medicine, University of Pennsylvania, Philadelphia, Pennsylvania, United States of America; 2 Centre for Eye Health, University of New South Wales, Kensington, Australia; 3 Department of Small Animal Clinical Sciences, College of Veterinary Medicine, Michigan State University, East Lansing, Michigan, United States of America; 4 Department of Ophthalmology, University of Florida, Gainesville, Florida, United States of America; Justus-Liebig-University Giessen, Germany

## Abstract

Mutations in the *BEST1* gene constitute an underlying cause of juvenile macular dystrophies, a group of retinal disorders commonly referred to as bestrophinopathies and usually diagnosed in early childhood or adolescence. The disease primarily affects macular and paramacular regions of the eye leading to major declines in central vision later in life. Currently, there is no cure or surgical management for *BEST1*-associated disorders. The recently characterized human disease counterpart, canine multifocal retinopathy (*cmr*), recapitulates a full spectrum of clinical and molecular features observed in human bestrophinopathies and offers a valuable model system for development and testing of therapeutic strategies. In this study, the specificity, efficiency and safety of rAAV-mediated transgene expression driven by the human VMD2 promoter were assessed in wild-type canine retinae. While the subretinal delivery of rAAV2/1 vector serotype was associated with cone damage in the retina when *BEST1* and GFP were co-expressed, the rAAV2/2 vector serotype carrying either GFP reporter or *BEST1* transgene under control of human VMD2 promoter was safe, and enabled specific transduction of the RPE cell monolayer that was stable for up to 6 months post injection. These encouraging studies with the rAAV2/2 vector lay the groundwork for development of gene augmentation therapy for human bestrophinopathies.

## Introduction

Disease-causing mutations in retinal pigment epithelium (RPE) and photoreceptor specific genes are a major cause of vision impairment worldwide. This is particularly devastating when the genetic alteration leads to a phenotype that affects foveal vision early in life and there are no treatment options available. Affected children and adolescents gradually lose central visual acuity that seriously impairs their quality of life in adulthood. Bestrophinopathies, both the autosomal dominant (Best Vitelliform Macular Dystrophy, BVMD) and autosomal recessive (Autosomal Recessive Bestrophinopathy, ARB) forms, belong to this group of disorders. Caused by *BEST1* (aka *VMD2*) mutations, this juvenile-onset macular degeneration, characterized by an abnormal RPE-photoreceptor interface and depressed EOG light peak, results in poor vision evident during the later stages of the disease [1]–[3]. To date, there is no specific treatment or surgical management for *BEST1*-related retinopathies.

The *BEST1* gene product, bestrophin1, is a multifunctional protein associated with the basolateral plasma membrane of the RPE where it primarily acts as an anion channel and regulator of intracellular calcium signaling [4]–[7]. To date, nearly 200 disease-causing mutations have been identified in human *BEST1* (h*BEST1*), and the *in vitro* analysis of their molecular and functional consequences revealed a total loss of Best1 channel activity in ARB or altered anion conductance due to defective intracellular trafficking and protein folding in BVMD [3], [8]–[10]. However, some aspects of the disease etiology and molecular pathology remain controversial [8]–[9]. Taking into account the considerably variable expressivity of *BEST1*-associated phenotypes and the fact that only a small percentage of *BEST1* mutations have been carefully studied, other unrecognized molecular mechanisms might contribute to the bestrophinopathy phenotypic spectrum. Thus, identification and development of a reliable animal model is critical to conduct translational research and address major unsolved questions in the disease pathogenesis.

Canine multifocal retinopathy (*cmr*), a spontaneous, early-onset autosomal recessive disease caused by mutations in the *BEST1* dog ortholog, recapitulates the full spectrum of clinical, genetic and histological features observed in *BEST1*-affected patients [11]–[14]. The *cmr* disorder results from any of the three distinct mutations identified to date in canine *BEST1* (c*BEST1*) that model all major aspects of known disease-associated mutations and their molecular consequences described in man: *cmr1* (C_73_T/R_25_X), an early stop mutation resulting in Best1 null phenotype; *cmr2* (G_482_A/G_161_D), a missense change affecting protein folding and trafficking; and *cmr3* (C_1388_del/P_463_fs), a frameshift mutation truncating the bestrophin1 C-terminus [11]–[14]. Since its discovery, *cmr* has been recognized as an important animal model for human bestrophinopathies suitable not only for carrying out mechanistic studies, but also for development and testing of therapeutic strategies such as recombinant adeno-associated virus (rAAV) based gene augmentation therapy.

Over the last decade, rAAV vectors have been proven safe in ocular tissue, and their versatility increased their use as gene delivery tools. Successful outcomes from the first clinical trials in patients affected with *RPE65* form of LCA were reported and have shown safety and efficacy [15]–[18]. While the rAAV2/2 serotype is the most widely used rAAV vector in preclinical and clinical trials when targeting the RPE, the rAAV2/1 serotype, which has also been shown to target this cell population [19], is thought to be more RPE-specific [20]. In preparation for gene augmentation therapy in *cmr*-affected dogs that could ultimately be translated to human *BEST1*-associated disorders, we compared the rAAV2/1 and rAAV2/2 vector serotypes carrying GFP reporter or *BEST1* transgene under control of human VMD2 promoter (hVMD2). The onset, efficiency and stability of transgene expression, as well as the potential adverse effects of a single subretinal injection with either vector serotype, were evaluated in wild-type canine eyes. Our findings provide evidence that the rAAV2/1-mediated delivery of the *BEST1* transgene potentially can cause cone-selective damage in the transduced retina. In contrast, the rAAV2/2 vector serotype is safe, specific, stable, and should be considered further for developing gene augmentation therapies in bestrophinopathies.

## Results

**Figure 1:**
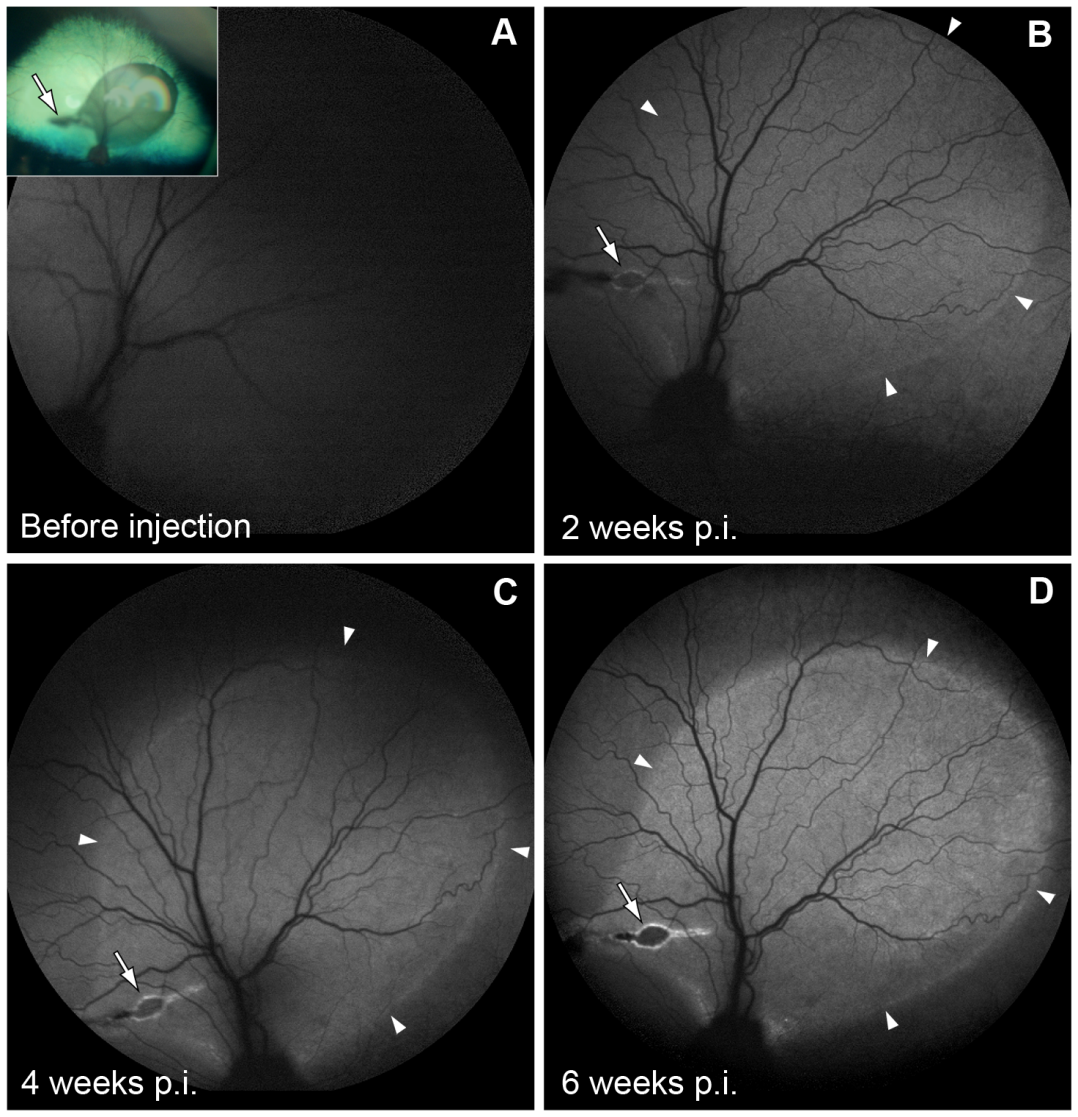
Recombinant AAV-mediated GFP expression in the canine eyes by fundus autofluorescence imaging. A representative summary of findings from rAAV2/2-hVMD2-GFP injected canine eyes. A normalized autofluorescence mode was used to obtain a baseline photograph for the native eye before vector administration (**A**). GFP fluorescence was first detectable at 2 weeks p.i. (**B**, arrowheads) and the outline of the injection area corresponded to the subretinal bleb documented immediately after vector administration (**A**, inset). An evident increase in GFP expression was observed at 4 to 6 weeks p.i. (**C, D**), when the peak of rAAV vector expression is expected. The boundaries of the autofluorescent areas (arrowheads), as well as retinotomy scar (arrows) remained unchanged throughout the observation period. High-resolution images were captured in AF mode at 55°; p.i.: post injection.

A set of eighteen eyes from twelve dogs was used to determine the cell specificity and level of rAAV-mediated GFP expression regulated by the hVMD2 promoter in the normal canine retina. The rAAV2/1 or rAAV2/2 vector serotypes encoding GFP reporter gene under control of hVMD2 promoter (Fig. S1A) were delivered by subretinal injection (Fig. 1, Tab. S1). Immediately after vector administration, formation of the characteristic bleb underlying the neural retina was observed, delimiting the injected area of the fundus (Fig. 1 insert). In all cases, the retinal detachment completely resolved within 24 hours post injection (p.i.), and only the focal retinotomy scar remained visible. By autofluorescence imaging, GFP expression was first detectable at 2 weeks p.i., and reached its expression peak between 4 – 6 weeks p.i. (Fig. 1). The results obtained for rAAV2/1-hVMD2-GFP and rAAV2/2-hVMD2-GFP were comparable and clinical assessments throughout the injection-endpoint evaluation period did not reveal any abnormalities with either vector construct in any of the injected eyes.

**Figure 2:**
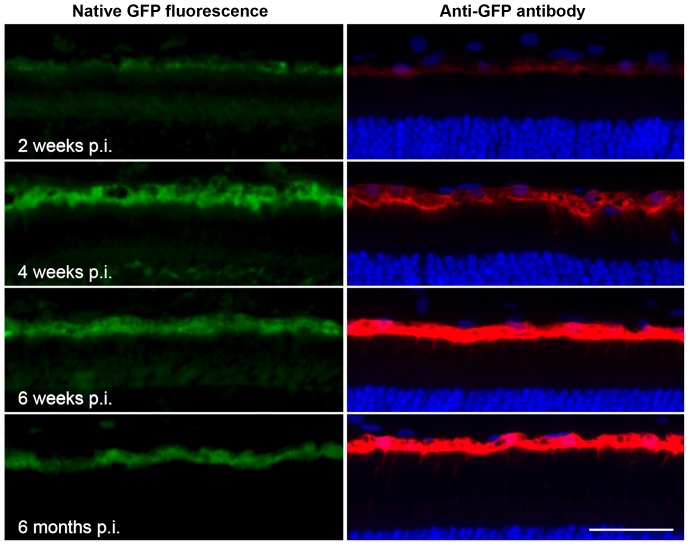
Specificity and stability of rAAV-mediated RPE transduction regulated by hVMD2 in the canine retina. The rAAV2/2 vector construct carrying GFP reporter gene under control of human VMD2 promoter specifically and exclusively target transgene expression to the RPE cells. Native (green) or anti-GFP probed (red) GFP expression was analyzed on frozen retinal cross-sections 2- (9.11×10^10^ vg), 4- (1.21×10^11^ vg), 6- (9.11×10^10^ vg) weeks, and 6 months (9.11×10^10^ vg) post injection. Immunohistochemical staining confirmed the gradual increase of the transgene expression level over the first 6 weeks p.i. that remained stable up to 6 months after vector administration. DAPI stain was used to detect cell nuclei; vg: vector genomes injected; p.i.: post injection; scale bar: 40 µm.

**Figure 3:**
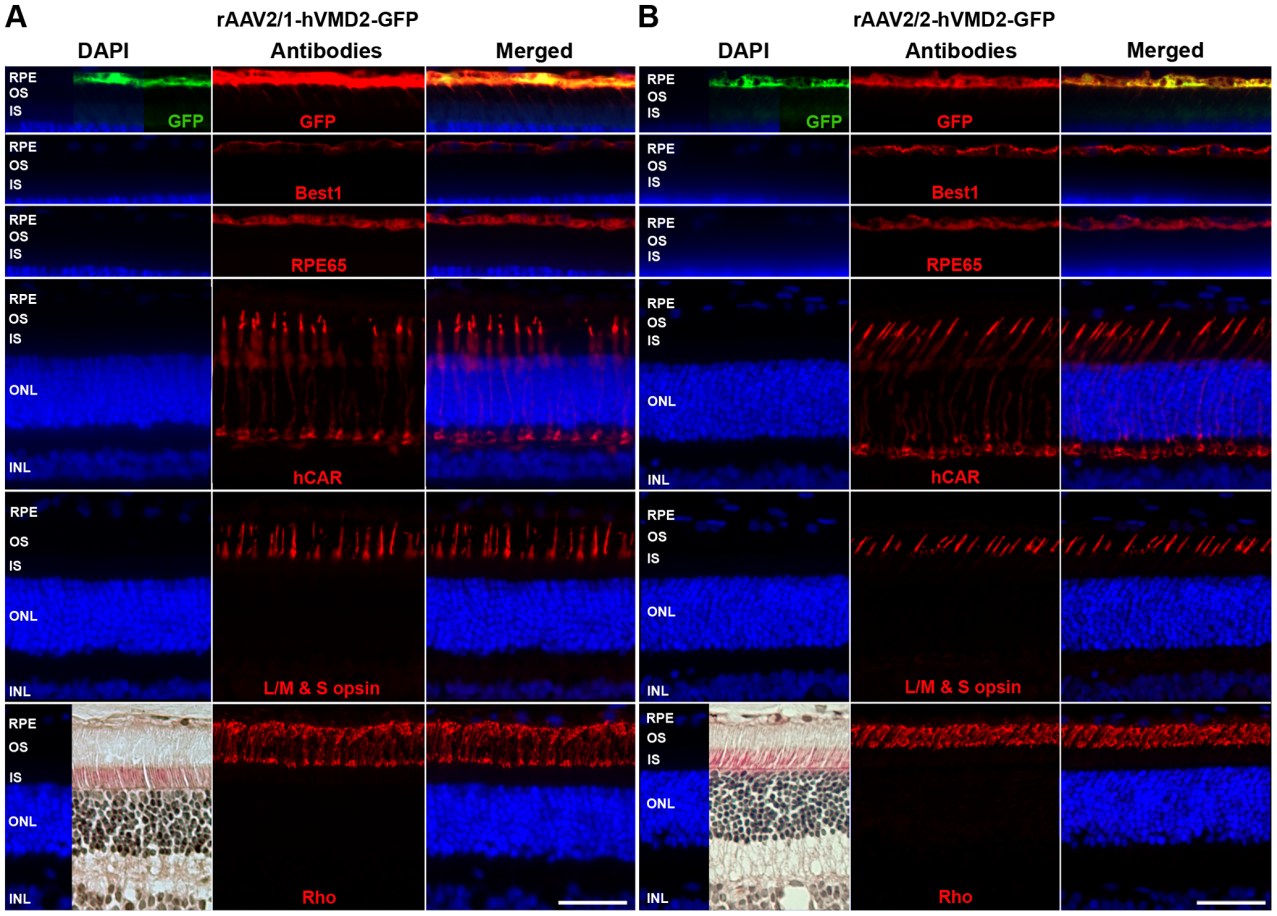
Comparison of rAAV2/1- and rAAV2/2-mediated GFP expression in the wild-type canine retina. Immunohistochemical assessment of rAAV2/1-hVMD2-GFP (2.63×10^11^ vg) (**A**) and rAAV2/2-hVMD2-GFP (9.11×10^10^ vg) (**B**) injected retinas 6 weeks post injection. GFP expression (native expression  =  green; anti-GFP antibody  =  red) is shown only in the first row of images; selected retinal and RPE proteins were evaluated by antibody labeling. RPE cells expressed Best1 and RPE65 proteins; the structure of cone photoreceptors was demonstrated by hCAR and L/M & S opsin labeling, while rods were assessed based on Rho localization. In all cases, protein expression was normal, specific and comparable to the non-injected eyes (data not shown), irrespective of the recombinant vector serotype used. Preservation of the retinal structure is demonstrated by H&E. RPE: retinal pigment epithelium, OS: photoreceptor outer segments; IS: photoreceptor inner segments; ONL: outer nuclear layer; INL: inner nuclear layer; cell nuclei were stained with DAPI; vg: vector genomes injected; scale bar: 40 µm.

Under control of the hVMD2 promoter, both vector serotypes demonstrated specific and exclusive cellular tropism for the RPE cell monolayer where the GFP reporter expression was detectable as native green fluorescence that colocalized with specific anti-GFP antibody labeling (Figs. 2, 3 and S2). In all cases, the extent of GFP expression in the RPE corresponded to the vector bleb area recorded immediately after subretinal injection. The gradual increase of GFP expression levels over the first 6 weeks p.i., and its stability up to 6 months after vector administration, were confirmed by immunohistochemical staining (Fig. 2). When taking into account the differences in total vector genomes injected, the transduction efficiency was qualitatively comparable between rAAV2/1 and rAAV2/2 serotypes (Fig. S2). Structural preservation of the retina was assessed by immunocytochemical staining 6 weeks p.i. using several RPE (Best1, RPE65) and cone (hCAR, L/M&S opsin) and rod (Rho) specific markers (Fig 3). In all instances, retinal structure was normal, and showed no damage to the RPE or photoreceptor cells based on immunolabeling and by H&E staining (Fig. 3A,B). Overall, there was no difference observed in the transduction efficiency, onset or stability between rAAV2/1 and rAAV2/2 serotypes carrying the reporter gene and, most importantly, no adverse effects were noted with either vector secondary to the subretinal injection. Together, these findings provided evidence that human VMD2 promoter drives stable and specific transgene expression to the RPE cells *in vivo* after single injection of rAAV2/1-hVMD2-GFP or rAAV2/2-hVMD2-GFP vectors.

Subsequently, a modified vector construct encoding full-length canine or human *BEST1* cDNA driven by the same promoter (Fig. S1B) was used to test rAAV-mediated *BEST1* transgene expression in wild-type canine retinae. The vector dosage was 7.10×10^10^–2.00×10^12^ vg and 3.92×10^11^–1.00×10^12^ vg injected for rAAV2/1-hVMD2-*BEST1* and rAAV2/2-hVMD2-*BEST1*, respectively (Tab. S1). In addition, to monitor the bleb kinetics and the spatial extent of a single subretinal injection, the rAAV2/1-hVMD2-*BEST1* vector constructs were spiked with the corresponding GFP expressing vector at an average titer of 2.5×10^9^ vg/ml (Tab. S1, Fig S3).

**Figure 4:**
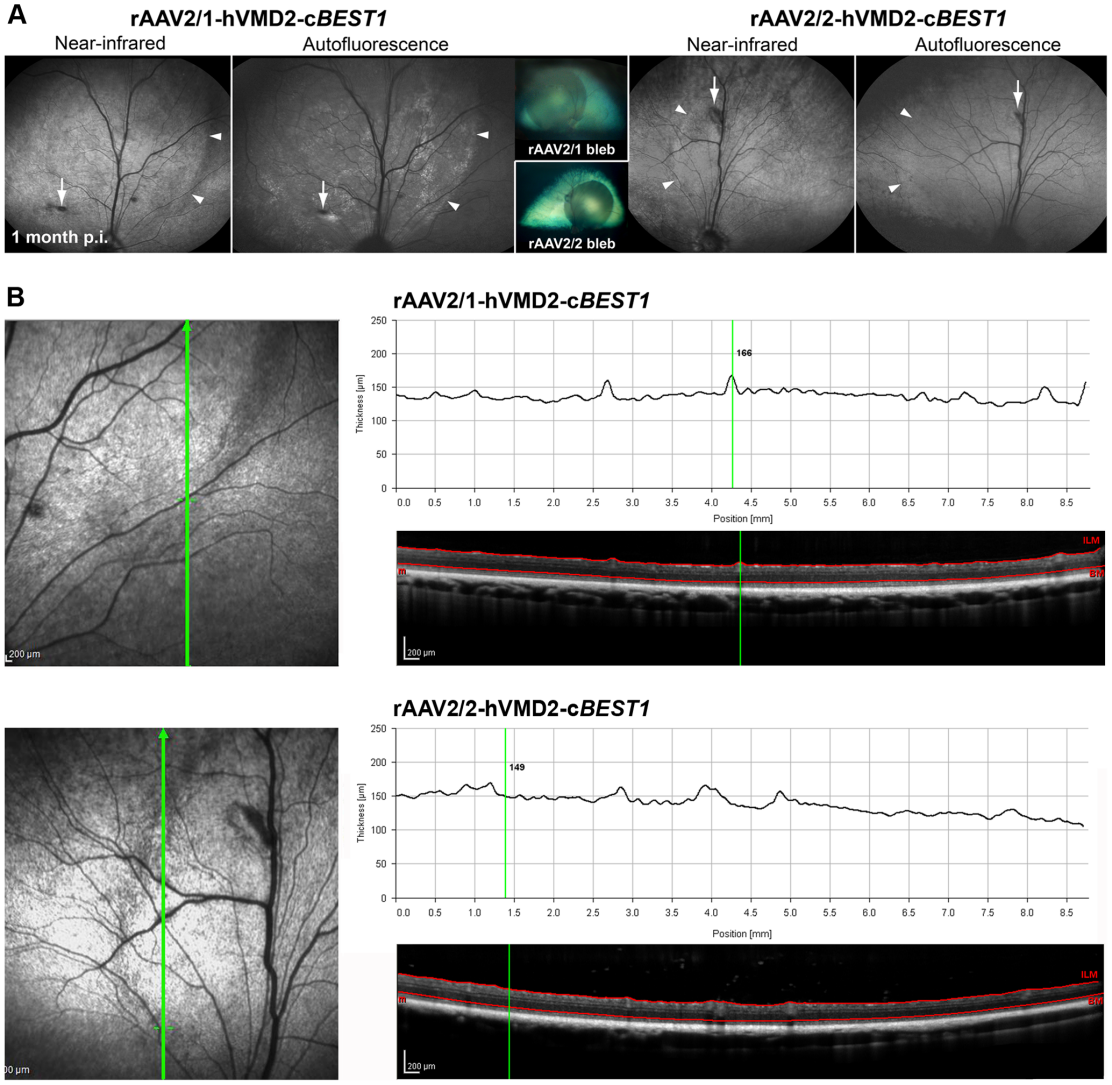
Evaluation of rAAV2/1- and rAAV2/2-mediated *BEST1* transgene expression in the canine retina. (**A**) Comparison of two normal canine eyes that received subretinal injection of rAAV2/1 (1.94×10^11^ vg) and a spike-in of corresponding vector expressing GFP (3.81×10^9^ vg) or rAAV2/2 (3.92×10^11^ vg) expressing canine *BEST1* under control of human VMD2 promoter. The outlines of the injected areas detectable in NIR mode and more evident in AF mode for rAAV2/1 (arrowheads) corresponded to the bleb formed immediately after injection (insets). The arrows indicate retinotomy sites. (**B**) Retinal thickness profiling done by manual segmentation across the bleb boundaries revealed no significant changes 4 weeks p.i. with either vector construct. High-resolution OCT images were obtained using a 30° lens; NIR and AF images were captured using a 55° lens; vg: vector genomes injected; p.i.: post injection.

For this study, a total of 11 normal eyes from eleven control dogs were subretinally injected with rAAV2/1 or rAAV2/2 carrying wild-type either canine or human *BEST1* and evaluated at several time points p.i. (Tab. S1). Similar to the prior reporter gene studies, the subretinal injection bleb resolved within 24 hours p.i., and the corresponding bleb boundaries were detectable by near-infrared reflectance imaging up to 4 weeks after vector administration (Fig. 4A). For the rAAV2/1-GFP-spiked injections, discrete areas of fluorescence were clearly visible in the autofluorescence mode over a 6 week monitoring period (Figs. 4A, S3). Again, all eyes used in this phase of experiment appeared clinically asymptomatic after the retinal reattachment and no obvious changes were noted throughout the observation period by routine fundus examination. The *in vivo* OCT imaging did not reveal changes in the retinal structure for any of the injected eyes, and, with either vector; the retinal thickness profiles were normal, and not different between the injected and non-injected regions (Fig. 4B).

**Figure 5:**
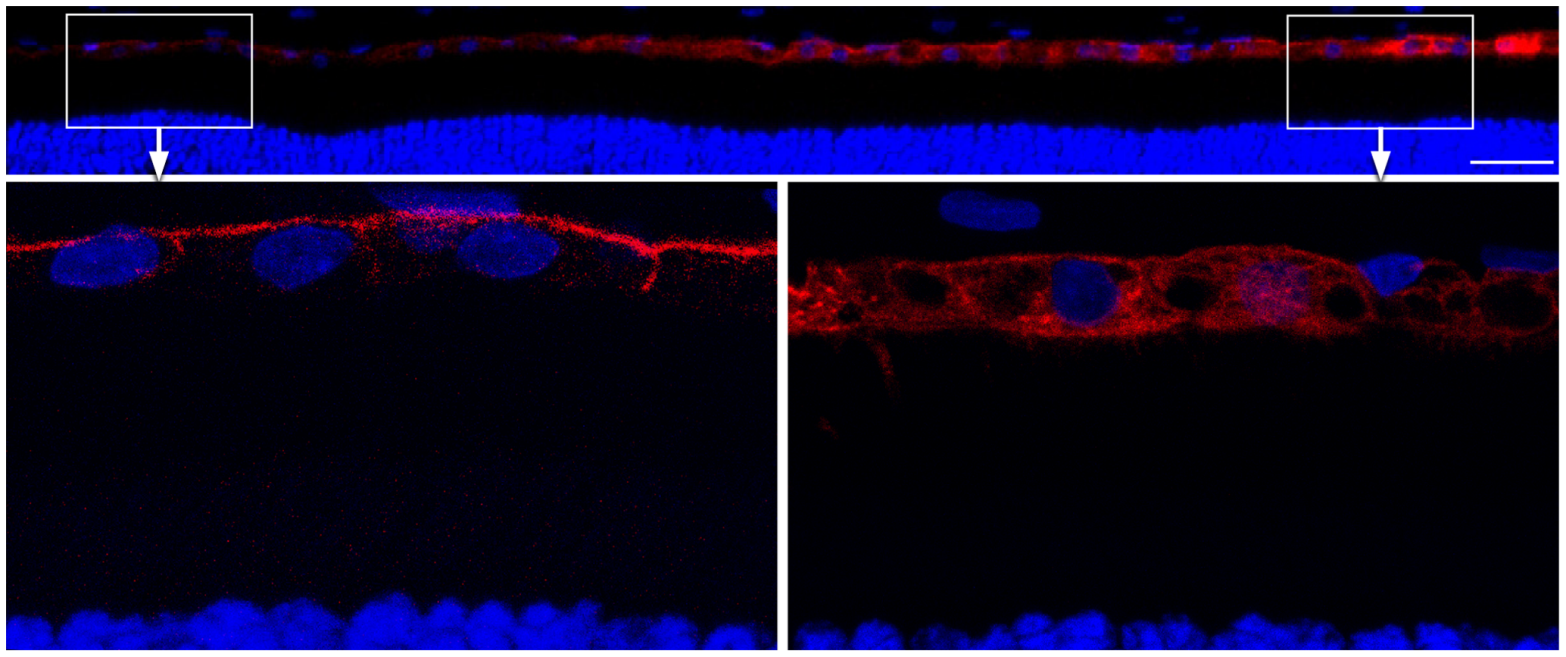
Bestrophin1 overexpression induced by rAAV2/2 in the wild-type canine retina. Confocal photomicrographs illustrating Best1 expression (red) in the wild-type canine RPE six months p.i. The endogenous expression of Best1 (boxed area left and corresponding magnification) was limited to the basolateral plasma membrane while the transgene protein was also observed in the cell cytoplasm as a result of overexpression mediated by rAAV2/2-hVMD2-c*BEST1* (3.92×10^11^ vg) (boxed area right and corresponding magnification). Cell nuclei were stained with DAPI; vg: vector genomes injected; p.i.: post injection; scale bar: 40 µm.

**Figure 6:**
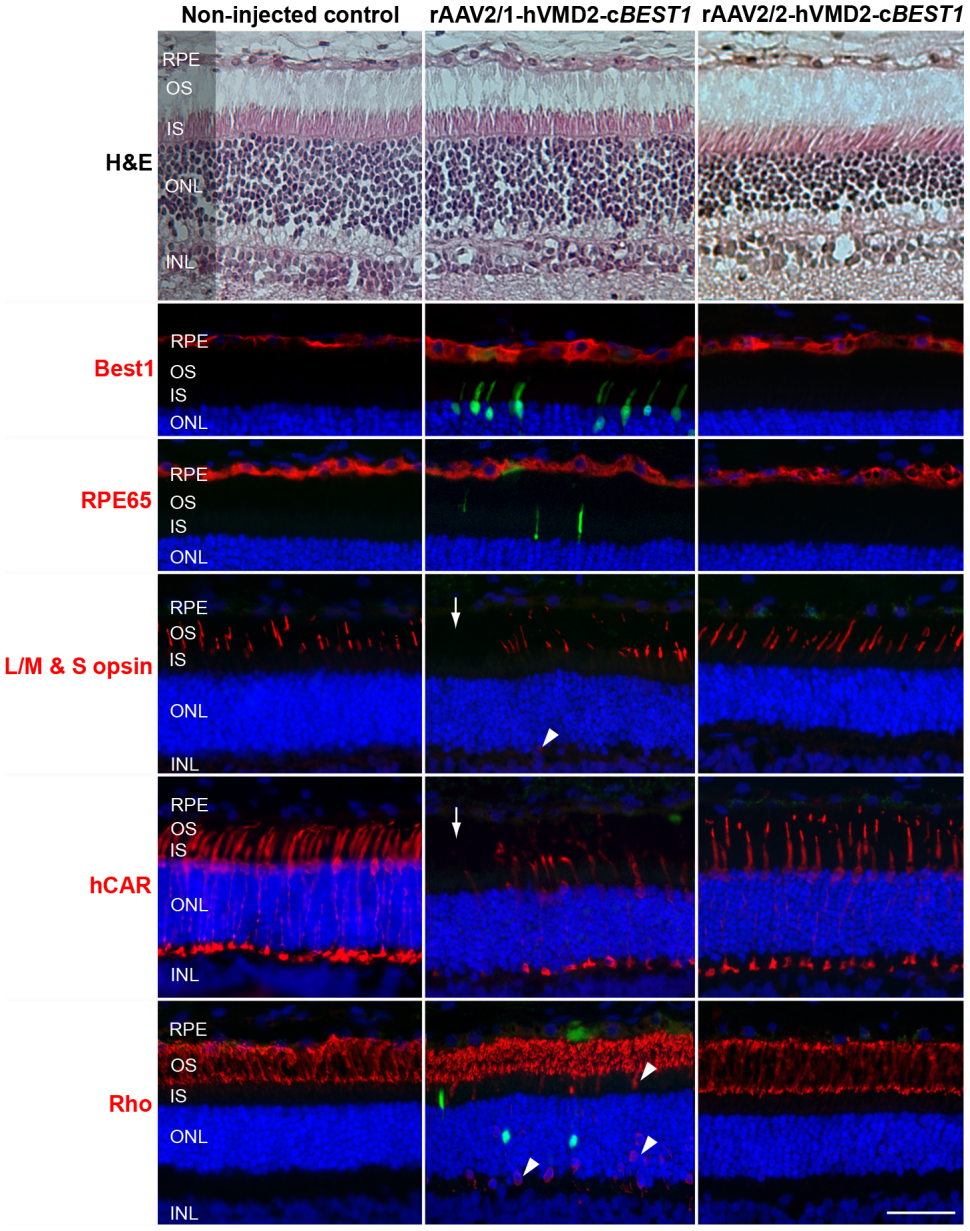
Consequences of rAAV2/1- and rAAV2/2-induced *BEST1* transgene expression *in vivo*. Histological and immunohistochemical evaluation of wild-type canine retinae injected with rAAV2/1-hVMD2-c*BEST1* (2.63×10^11^ vg) and a spike-in of corresponding vector expressing GFP (2.5×10^9^ vg) or rAAV2/2-hVMD2-c*BEST1* (4.44×10^11^ vg) in comparison to the non-injected control. H&E staining did not reveal any histological changes with either vector serotype. Both vectors induced bestrophin1 overexpression in the RPE cells 4 weeks post injection (Best1, red). While no abnormalities were observed in rAAV2/2-transduced retina, the rAAV2/1 serotype caused fluorescence in individual photoreceptor cells (green), occasional mislocalization of cone and rod opsins (arrowheads) and patchy loss of cone photoreceptors (arrows) in the rAAV2/1-hVMD2-c*BEST1*-injected area. RPE: retinal pigment epithelium, OS: photoreceptor outer segments; IS: photoreceptor inner segments; ONL: outer nuclear layer; INL: inner nuclear layer. Cell nuclei were stained with DAPI; vg: vector genomes injected; scale bar: 40 µm and applies to all panels.

The rAAV-mediated c*BEST1* transgene expression in the normal canine retina was evaluated 4- and 6 weeks and 6 months p.i. (Tab. S1). While the endogenous expression of Best1 was limited to the basolateral plasma membrane, the transgene protein - found only in the bleb area - was localized within the RPE cell cytoplasm as a result of vector mediated overexpression; the results were the same with either vector (Fig. 5 illustrates the findings with rAAV2/2). The consequences of rAAV2/1- and AAV2/2-induced c*BEST1* expression in the normal retina were compared at 4 weeks p.i., and retinal preservation was assessed by H&E staining and cell-specific immunolabeling (Fig. 6). Even though the H&E staining did not indicate any morphological abnormalities with either vector serotype, specific immunolabeling and fluorescence microscopy of the rAAV2/1-hVMD2-c*BEST1*-transduced regions revealed green autofluorescence in individual photoreceptor cells, patchy to generalized loss of cone photoreceptors and mislocalization of both cone and rod opsins in treated areas (Fig. 6). Mislocalized rod opsin was seen in the inner segments (IS), outer nuclear layer (ONL) and in the outer plexiform layer, whereas the misrouted L/M & S opsins in some of the remaining cones were detected only in the ONL of rAAV2/1-hVMD2-c*BEST1*-injected areas (Fig. 6). In contrast, bestrophin1 expression mediated by the rAAV2/2-hVMD2-c*BEST1* construct did not result in any photoreceptor, RPE or retinal abnormalities (Fig. 6). There was no evidence of any inflammatory response in any of the injected eyes.

**Figure 7:**
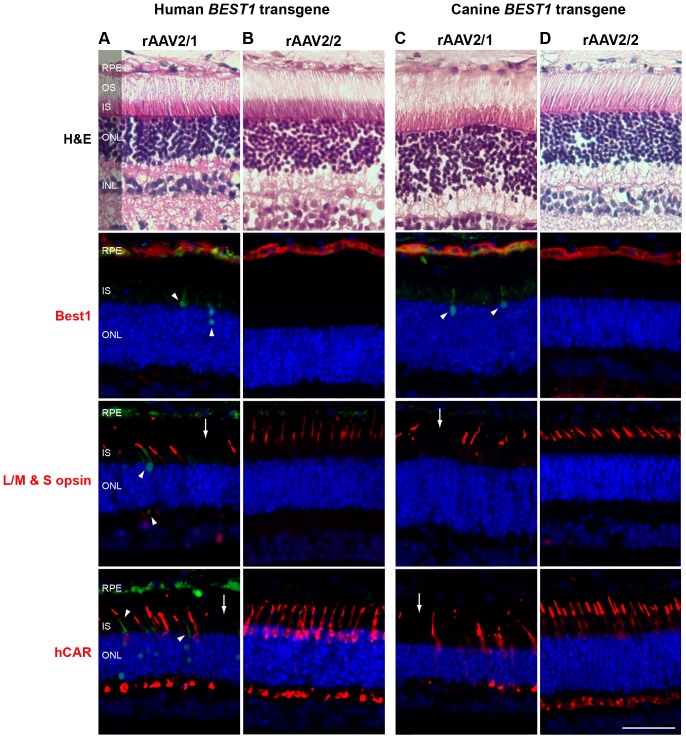
rAAV-mediated *BEST1* transfer to the retinal pigment epithelium and cone toxicity associated with rAAV2/1 vector serotype. (A-B) Co-expression of endogenous canine and human Best1 transgene *in vivo*. Histological and immunohistochemical analysis of wild-type canine retinae injected with rAAV2/1-hVMD2-h*BEST1* (1.16×10^12^ vg) and a spike-in of corresponding vector expressing GFP (1.04×10^9^) **(A)** and rAAV2/2-hVMD2-h*BEST1* (8.82×10^11^ vg) **(B)** at 4 weeks post injection. No structural abnormalities were seen by H&E staining in any of the samples. In the retina transduced with rAAV2/1 capsid serotype (A), individual photoreceptor cells emitted green autofluorescence (arrowheads) as shown on the photomicrographs probed with anti-Best1, anti-L/M & S opsin and anti-hCAR (red) (A). Cone-specific labeling revealed loss of cone photoreceptors (arrows) only in the areas transduced with rAAV2/1 vector serotype **(A)**. Co-expression of endogenous canine bestrophin1 and human *BEST1* transgene was well tolerated when injected with rAAV2/2 serotype and no abnormalities were noted **(B)**. **(C–D)** Comparison of rAAV2/1- and rAAV2/2-mediated c*BEST1* transgene expression in the *cmr1* (C_73_T/R_25_X) carrier retina. Histological and immunohistochemical evaluation of *cmr1* ^+/−^ retinae injected with rAAV2/1-hVMD2-c*BEST1* (1.92×10^11^ vg) and a spike-in of corresponding vector expressing GFP (1.74×10^9^) **(C)** and rAAV2/2-hVMD2-c*BEST1* (4.44×10^11^ vg) **(D)** at 4 weeks post injection. No morphological abnormalities were detected by H&E staining between the two capsid serotypes **(C–D)**. Sporadic autofluorescent cells (green) were observed in IS and ONL layers (**C**, arrowheads), and cone-specific immunolabeling (L/M & S opsin and anti-hCAR in red) revealed focal loss of cone photoreceptors (arrows) within the rAAV2/1-hVMD2-c*BEST1*-injected area **(C)**. rAAV2/2-mediated c*BEST1* transfer to the *cmr1* ^+/−^ retina results in bestrophin1 overexpression in the RPE (**D**, Best1 in red) with no adverse effects in the retinal tissue **(D)**. RPE: retinal pigment epithelium, OS: photoreceptor outer segments; IS: photoreceptor inner segments; ONL: outer nuclear layer; INL: inner nuclear layer. Cell nuclei were stained with DAPI; vg: vector genomes injected; scale bar: 40 µm and applies to all panels.

Furthermore, co-expression of the canine endogenous and human *BEST1* transgene was tested in the wild-type dog retina and evaluated at 4- and 6 weeks post injection. Both vector serotypes resulted in Best1 transgene expression in the RPE monolayer at 4 weeks p.i. (Fig. 7A,B). Again, whilst the co-expression of endogenous canine and human bestrophin1 transgene is well tolerated when mediated by rAAV2/2 serotype (Fig. 7B), introduction of rAAV2/1-hVMD2-h*BEST1* vector construct with a low level of corresponding vector expressing GFP resulted in patchy to generalized loss of cones and rather robust green fluorescence emitted by individual photoreceptor cells that, presumably, were damaged (Fig 7A). There were no RPE/photoreceptor/retinal alterations found in the rAAV2/2-hVMD2-h*BEST1*-injected eyes at 4- or 6 weeks p.i. (Fig. 7B).

We then determined whether the RPE/retinal abnormalities observed with rAAV2/1-hVMD2-*BEST1* were vector dose-dependent. To this end, the two vector serotypes carrying c*BEST1* were administered to the *cmr1* (C_73_T/R_25_X) carrier retinae that express half the normal levels of endogenous Best1 [13] (Fig. 7C,D). Delivery of rAAV2/1-hVMD2-c*BEST1* (7.10×10^10^ – 1.92×10^11^ vg) or rAAV2/2-hVMD2-c*BEST1* (4.44×10^11^ vg) still resulted in bestrophin1 overexpression in *cmr1* ^+/−^ model at 4 weeks p.i. (Fig. 7C,D). Similar to the prior experiments with rAAV2/1, absence or reduced number of cones was observed in the rAAV2/1-hVMD2-c*BEST1*-injected region, albeit the routine H&E staining did not indicate structural damage to the tissue (Fig. 7C). In contrast, the *cmr1* ^+/−^ retina transduced with rAAV2/2 serotype showed no adverse effects (Fig. 7D).

## Discussion

Progressive loss of central vision due to mutations in the *BEST1* gene is one of the most common causes of hereditary early macular degeneration [21]. Despite the fact that this ocular condition was first described over a century ago [22], the causal mechanism underlying its pathology is still poorly understood, and no cure is available. Here, we report studies on rAAV-mediated transgene expression using the human bestrophin promoter (hVMD2) in a large animal model as a first step towards developing gene augmentation therapy for human bestrophinopathies.

Although the stability and safety of rAAV2/2-mediated transgene expression in the canine retina has been previously assessed [19], [23] and successfully translated to the human subjects (for review see [18]), this is the first study using rAAV2/1 and rAAV2/2 vectors incorporating the hVMD2 promoter to evaluate their potential use in RPE-directed gene therapy. Initially, the specificity, stability and safety of the constructs carrying GFP reporter gene controlled by the hVMD2 promoter were tested in the wild-type canine retina. A single subretinal injection of rAAV2/1-hVMD2-GFP or rAAV2/2-hVMD2-GFP specifically and exclusively targeted transgene expression to the RPE cells, and no significant difference in onset, expression level or stability was observed between the two serotypes. Most importantly, no adverse effects were noted, either in the RPE or neuroretina, with either vector construct secondary to the subretinal injection.

Following these encouraging results from the pilot study, the efficiency and *BEST1* transgene expression was compared between the rAAV2/1 and rAAV2/2 serotypes. A dose range of 7.10×10^10^ – 2.00×10^12^ vg injected was tested in a set of 11 normal canine eyes delivering either canine or human *BEST1* transgene. In each case, introduction of *BEST1* into the wild-type retinae induced bestrophin1 expression limited to RPE cells of the injected area. The eyes did not demonstrate any clinical abnormalities, and all injected areas appeared histologically normal with H&E staining. There was no inflammatory response observed in any of the injected eyes. However, rAAV2/1 injected eyes with either human or canine *BEST1*, and containing low-level of corresponding vector expressing GFP reporter gene, revealed numerous photoreceptor cells, and, as expected, some RPE cells, that emitted green fluorescence. Based on their morphology and nuclear position, we interpreted the majority of the fluorescent photoreceptors to be cones. While this was confirmed, unexpectedly, we also found with photoreceptor specific immunolabeling that in all rAAV2/1-hVMD2-*BEST1*-treated areas there was a patchy to generalized loss of cones as well as mislocalization of cone and rod opsins. The loss of cones observed in all rAAV2/1-hVMD2-*BEST1*-injected regions was evident by lack of hCAR and L/M&S cone opsins immunoreactivity, as well as absence or reduced number of cone photoreceptor cells visualized by Nomarski DIC microscopy (data not shown). Such photoreceptor toxicity was not observed when using the rAAV2/2 vector at the same dosage level, or when using comparable doses of either rAAV2/1 or rAAV2/2 expressing only the GFP transgene and regulated by the hVMD2 promoter. We previously have found cone-specific damage in the canine retinae transduced with high doses (1.51–4.79×10^13^ vg/ml) of rAAV2/5-GFP vectors with the hGRK1 or CBA promoters [24]. These vector doses, however, were ∼10,000 fold higher than the doses of GFP containing vector used to delimit the injected areas.

To exclude a vector-dose effect, the two vector serotypes were evaluated in asymptomatic *cmr1* (C_73_T/R_25_X) carrier retinae that express only half the normal levels of endogenous Best1 [13]. The extent of cone-selective damage caused by rAAV2/1 transduction was comparable to that detected in the wild-type tissue. It is not clear at this time whether the damage and loss of cones was a direct effect, i. e. resulted from transduction of cones with the vector, or an undefined indirect effect via RPE transduction. The interaction between rAAV2/1-hVMD2-*BEST1* and the rAAV2/1-GFP reporter construct cannot be ruled out, although the rAAV2/1-hVMD2-GFP vector construct by itself proved to be non-toxic to the retina. Furthermore, the effect of rAAV2/1 serotype capsid and its interaction with cell surface receptors cannot be eliminated either and might influence transduction pattern and, consequently, the delicate microenvironmental balance of the interphotoreceptor matrix. Considering that transgene expression levels are dose-dependent, and the physiological level of Best1 expression in the wild-type RPE cells are relatively low and limited to the basolateral plasma membrane, determination of a lower, safer and effective dosages of rAAV2/1-hVMD2-*BEST1*, without GFP admixture, may be important for future analysis. Because of the observed rAAV2/1-hVMD2-*BEST1*-associated cone toxicity this vector-promoter combination was not evaluated further in the present study. However, it will be a subject of more in depth future investigations.

It important to consider the cone-selective damage found in the canine retina in the context of future pre-clinical safety studies. While non-human primates or pigs have very distinct cones that are readily visualized by conventional histopathology in H&E stained sections, cones in dogs, mice and rats are difficult to identify, and damage to these cells, in the absence of a generalized photoreceptor atrophy, may go unrecognized. For that reason, we would suggest that specific immunolabeling of cones should be incorporated in safety assessments.

This work provides evidence that bestrophin1 overexpression mediated by rAAV2/2 serotype has no adverse effects on the RPE or retina, and *BEST1* transgene expression is RPE-specific and stable. There was no evidence of retinal toxicity in the four eyes that have been followed for 4–6 weeks post injection. Additionally, one wild-type eye treated with c*BEST1* transgene was followed for 6 months p.i. and also showed no evidence of toxicity. These preliminary results suggest that Best1 overexpression in the RPE results in no adverse effects. However, ongoing long-term studies on rAAV2/2-hVMD2-*BEST1*-mediated overexpression need to be concluded. Our findings to date strongly suggest that this approach is safe and might be useful in a mutation-independent basis. Thus, gene augmentation alone might be applied to all autosomal recessive and to those autosomal dominant mutations resulting in protein haploinsufficiency [25]. It is still to be proven whether gene augmentation alone is effective in cases where a dominant negative effect is suspected or whether the combination of mRNA silencing/resistant replacement strategy might be necessary [26]–[27]. Surprisingly, gene augmentation alone has been used successfully in the canine model of X-linked retinitis pigmentosa caused by RPGRORF15 microdeletion that resulted from a putative toxic gain of function [25], [28]. Fortunately for bestrophinopathies it is possible to model the human and canine mutations *in vitro* and determine the optimal therapeutic approach [13].

Co-expression of canine endogenous and rAAV2/2-mediated human *BEST1* transgene is well tolerated in the wild-type retina. This strengthens the model system even more and will facilitate the translational studies of Best1 therapy in the future. Proof of concept studies in *cmr*-affected dogs are currently in progress.

Many canine diseases have clinical and molecular human counterparts [29]–[30]. The latest successes using rAAV-mediated gene augmentation therapy, first evaluated in large animal models [19], [23] and then translated to clinical use [15]–[18], have paved a way for considering recombinant AAV vectors for treatment of other human retinopathies previously considered untreatable. Our study on rAAV-mediated *BEST1* transgene expression in the canine RPE represents a first step towards gene augmentation therapy for bestrophinopathies. The unique combination of a spontaneous animal model on a stable genetic background with anatomical and phenotypic similarities to the human eye provides the exceptional opportunity to develop and test treatment options that can rapidly be translated to the clinic.

## Materials and Methods

### Animals

Twenty-nine eyes of young adult crossbred controls (23 dogs; n = 29 eyes) or three *cmr1* carriers (n = 5 eyes) were used in this study (Tab. S1). All animals were bred and maintained at the Retinal Disease Studies Facility (RDSF), Kennett Square, PA and supported by facility grants from FFB and NEI/NIH EY06855. The studies were carried out in strict accordance with the recommendations in the Guide for the Care and Use of Laboratory Animals of the National Institutes of Health, and in compliance with the ARVO Statement for the Use of Animals in Ophthalmic and Vision Research. The protocols were approved by the Institutional Animal Care and Use Committee of the University of Pennsylvania (IACUC Protocol #s 801870, 803422). All efforts were made to improve animal welfare and minimize discomfort.

### Vector production

The human VMD2 promoter driving “humanized” GFP reporter [31] or *BEST1* gene was evaluated in recombinant adeno-associated virus vector serotypes 1 (rAAV2/1) or 2 (rAAV2/2). Briefly, the hVMD2 promoter [32] (courtesy of D. J. Zack, Johns Hopkins University) was ligated into a recombinant AAV vector plasmid containing GFP or c*BEST1* or h*BEST1*, SV40 splice donor/acceptor sites and polyadenylation signal (Fig. S1). The resulting plasmid constructs, hVMD2-GFP, hVMD2-c*BEST1* or hVMD2-h*BEST1* were then packaged into rAAV using standard vector preparation methods [31], [33] and titered for DNase-resistant vector genomes by RT-PCR relative to a standard using oligonucleotides targeting the SV40 polyadenylation signal (Forward: 5′-TTT GTG AAA TTT GTG ATG CT-3′; Reverse: 5′-TGA ATG CAA TTG TTG TTG TT-3′). The reaction mix was set up using iQ SYBR® Green Supermix (Bio-Rad Laboratories, Hercules, CA) and performed in a MyiQ™ Real-Time PCR Detection System (Bio-Rad Laboratories, Hercules, CA). Titers were calculated using the MyiQ™ Optical System Software, (Bio-Rad Laboratories Hercules, CA). The standard used for the Real-Time PCR was a rAAV with a known titer that was independently verified by PCR, dot blot and Infectious Center Assay (Vector Core Facility, University of Florida). Finally, the purity of the vector was validated using three standard assays. First, by silver-stained SDS-PAGE to confirm presence of the three capsid proteins; secondly, by assay screening for bioburden by spreading 10 µl of the final product on a non-selective LB-agar plate; and lastly, the final product was assayed for endotoxins using the Endosafe-PTS, portable test system (Charles River Laboratories, Charleston, SC, USA). For the rAAV2/1-hVMD2-*BEST1* vectors with GFP spike-in, a portion of vector plasmid was used to package the GFP transgene. The amount of final GFP virus in the ad-mixture was determined by RT-PCR tittering with oligonucleotides targeted GFP cDNA. The average titer of the GFP component was 2.5×10^9^ vg/ml, with a range of 1.05×10^9^ to 3.81×10^9^ vg/ml. Viral vector stocks were kept at −80°C in Balance Salt Solution (BSS; Alcon, Fort Worth, TX, USA), and all subsequent dilutions were prepared using BSS.

### Subretinal injection and postoperative procedures

Subretinal injections of rAAV2/1 or rAAV2/2, carrying the GFP reporter or *BEST1* wild-type cDNA sequence (canine or human), were performed under general anesthesia following previously published procedures [24]–[25], [34]. A total volume of 90–200 µl of the viral vector solution, with different vector genome concentrations, was injected subretinally with a RetinaJect subretinal injector (SurModics Inc., Eden Prairie, MN, USA) under direct visualization with an operating microscope via a transvitreal approach without vitrectomy (Tab. S1). Each injection was directed to the superior temporal quadrant of the eye, a region with a uniform density of cone photoreceptors [35]. Fundus visualization was achieved with either a Machemer magnifying vitrectomy lens (OMVI; Ocular Instruments Inc., Bellevue, WA, USA), or a vitreoretinal surgery contact lens (AcrivetVit.Lens; Acrivet, Salt Lake City, UT, USA). Directly after injection, formation of a subretinal bleb was documented. An anterior chamber paracentesis was performed immediately after injection to prevent increase in intraocular pressure, followed by subconjunctival injection of 4 mg of triamcinolone acetonide.

Ophthalmic examinations, including biomicroscopy, indirect ophthalmoscopy and fundus photography, were conducted on a regular basis throughout the injection-endpoint evaluation time interval. Postoperative topical medication included application of atropine sulfate 1% ophthalmic ointment twice daily for 8 days, followed by once daily for 3 days; and neomycin/ polymyxin B sulfate/ dexamethasone ophthalmic ointment twice daily for 8 days, then once daily for 6 days. Systemic antibiotics (amoxicillin trihydrate/ clavulanate potassium 12.5 mg/kg) were given orally twice daily for 3 days, and prednisone tablets (1 mg/kg) were administrated twice daily for 9 days, followed by once daily for 1 week.

### In vivo retinal imaging

Imaging was performed under general anesthesia using a cSLO/sdOCT instrument (Spectralis™ HRA/OCT, Heidelberg, Germany) on a bi-monthly basis. *En face* imaging was done using near infrared mode (NIR) to register fundus changes, while the native GFP expression was documented using short-wavelength autofluorescence mode (AF); both modes with a 55° lens. Spectral-domain optical coherence tomography (sd-OCT) was performed with linear and raster scans using a 30° lens. Linear scans were placed across regions of interest and images were averaged (20 ART) and taken in a 30 degree area (line). Raster scans covered a 30×20 degree retinal areas with a 49 sequential B-scans, each one separated by 120 µm and with the average of 9 ART. The representative OCT scans were manually segmented to identify canine ELM and measure the ELM-ILM thickness.

### Immunohistochemistry

Histological assessment using standard H&E staining and immunohistochemical analyses were performed on 10 µm cryosections using standard protocols [24], [36]. Antibodies directed against GFP, Best1, RPE65, rod opsin, human cone arrestin, red/green cone opsin and blue cone opsin were used in single immunolabeling reactions and visualized with Alexa Fluor 568 nm goat anti-rabbit or goat anti-mouse secondary antibody; cell nuclei were stained with DAPI. Details of antibodies, dilution and source are in Table S2. Slides were mounted (Gelvatol, Sigma-Aldrich, St. Louis, MO, USA) and examined with by epifluorescence or transmitted light microscopy (Axioplan; Carl Zeiss Meditec).

### Confocal microscopy

Confocal images were acquired on a Leica TCS-SP5 tunable spectral confocal and multi-photon system with a Leica DM 6000 CFS upright microscope (Leica Microsystems, Wetzlar, Germany) through a HCX PL APO 40×(N.A. 1.25) or 60×(N.A. 1.40) oil immersion objectives. Alexa Fluor 568 was excited at 543 nm laser line and emission collected at 574–700 nm in sequential scanning mode by the tunable internal detectors. DAPI was excited with multi-photon at 750 nm produced by a Coherent Chameleon Ultra II Ti:sapphire pulse laser (Santa Clara, CA, USA) and emission was collected at 380–500 nm.

## Acknowledgments

The authors thank Karla Carlisle and the Retinal Disease Studies Facility staff for animal care, Emily Dutrow and Julianna Slavik for excellent technical assistance, Al S. Lewin (University of Florida) for helpful discussions and suggestions, W. Clay Smith (University of Florida) for anti-GFP antibody, C.M. Craft (University of Southern California) for anti-hCAR antibody, Mary Leonard (Biomedical Art & Design, University of Pennsylvania) for assistance in graphic design, and Lydia Melnyk for editorial help and research coordination.

## Supporting Information

Figure S1 **Schematic diagrams of plasmid constructs used for rAAV2-hVMD2-GFP and rAAV2-hVMD2-** ***BEST1*** **vectors production.** (**A**) Map of the pTR-VMD2-GFP plasmid used to produce the rAAV2/1-hVMD2-GFP and rAAV2/2-hVMD2-GFP vector constructs. (**B**). Map of the pTR-VMD2-*BEST1* plasmid used to produce the rAAV2/1-hVMD2-c*BEST1*, rAAV2/2-hVMD2-c*BEST1*, rAAV2/1-hVMD2-h*BEST1* and rAAV2/2-hVMD2-h*BEST1* vector constructs. TR: AAV2 inverted terminal repeats; VMD2: human VMD2 promoter [32]; SV40 SD/SA: SV40 late viral protein gene 16S/19S splice donor and acceptor signal; hGFP: “humanized” green fluorescence protein reporter^31^; BEST1: coding sequence of wild-type canine *BEST1* or wild-type human *BEST1* gene; SV40 (poly A) and bGH poly (A): polyadenylation signals; HSK-tk: thymidine kinase promoter of the herpes simplex virus; Neo R: coding sequence of the neomycin resistance gene.
(TIF)

Figure S2 **Transduction efficiency of rAAV2/1 and rAAV2/2 vectors carrying GFP reporter under control of human VMD2 promoter.** Comparison of GFP expression levels induced by rAAV2/1 (2.63×10^11^ vg) or rAAV2/2 (9.11×10^10^ vg) at 6 weeks p.i. Native GFP expression (green) appeared more pronounced in the rAAV2/1 transduced RPE cells as confirmed by a dilution series of anti-GFP antibody (red). Expression levels induced with rAAV2/1 were detectable up to 1∶75,000 dilution (left panel), while the rAAV2/2-mediated expression could not be visualized beyond a 1∶50,000 dilution (right panel). Considering the difference in total virus genomes injected, however, both vectors appear to be qualitatively similar in transduction efficiency and both specifically target transgene expression to the RPE cell monolayer. Cell nuclei were stained with DAPI; vg: vector genomes injected; p.i.: post injection; scale bar: 40 µm.
(TIF)

Figure S3 **Monitoring the bleb kinetics and spatial extent of single subretinal injection in the canine fundus.** (**A–D**) rAAV2/1-hVMD2-c*BEST1*-injected eye (1.94×10^11^ vg/ml) with a spike-in of corresponding vector expressing GFP (3.81×10^9^ vg/ml); a higher magnification figure of part of the fundus is shown in Fig. 4A. Composite fundus images NIR 55° 82°×56° (**A**) and AF 55° ART 98°x58° (**B**) captured 4 weeks after single subretinal injection of 150 µl. Note the GFP spiked area visible in autofluorescence mode (**B**) delimiting the spatial extent of injection (arrowheads). The arrow indicates retinotomy site. (**C–D**) Single (**C**) and double (**D**) immunolabeling of anti-GFP (green) and anti-hCAR (red). The GFP-positive cells scattered in the RPE monolayer corresponded to the injection boundaries outlined by AF mode (**B**). This area shows minimal damage to cones. Cell nuclei were stained with DAPI; scale bar: 40 µm.
(TIF)

Table S1 **Summary of injected subjects used in the studies.** Recombinant adeno-associated virus vectors and dosage analyzed in total of 34 canine eyes. Eyes #: number of eyes injected per each type of vector; vg: vector genomes injected; Evaluation time point: post injection time by endpoint evaluation. p.i.: post injection.
(DOCX)

Table S2 **List of primary antibodies used for immunolabeling.** Tissue sections were washed in 1XPBS/0.25% TX-100 for 5 minutes and blocked for 1 h (10% normal goat serum, 1XPBS/0.25% TX-100, 0.05% sodium azide). Overnight incubation (at 4°C) with antibodies listed in the table was followed by three 1XPBS washes and incubation with 1∶200 Alexa Fluor 568 nm goat anti-rabbit (A11036, Invitrogen, Carlsbad, CA, USA) or goat anti-mouse (A11031, Invitrogen, Carlsbad, CA, USA) secondary antibody, respectively, for 1 h at room temperature.
(DOC)
