# Supplementary figures and images for: Recombinant AAV-Mediated BEST1 Transfer to the Retinal Pigment Epithelium: Analysis of Serotype-Dependent Retinal Effects
Source: PLoS One. 2013 Oct 15;8(10):e75666. doi: 10.1371/journal.pone.0075666 (PMC3797066; doi:10.1371/journal.pone.0075666)

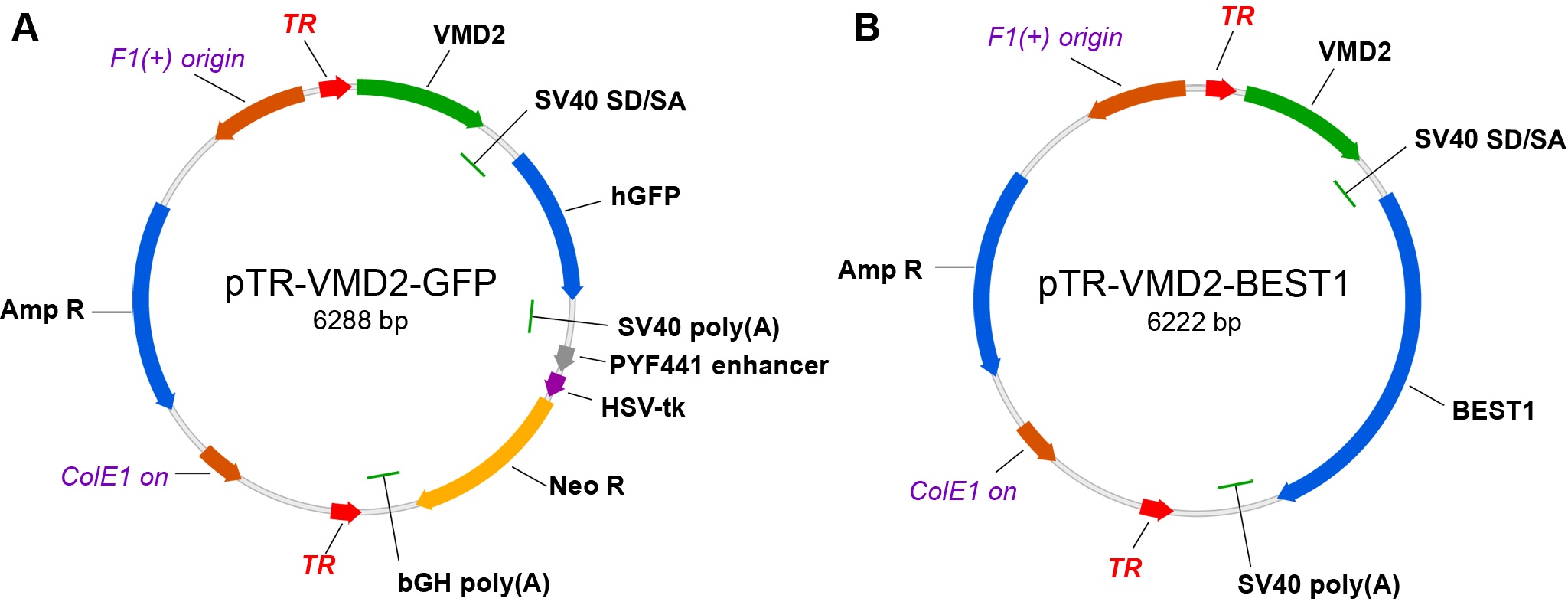

Supplement: Figure S1 — Schematic diagrams of plasmid constructs used for rAAV2-hVMD2-GFP and rAAV2-hVMD2- BEST1 vectors production. (A) Map of the pTR-VMD2-GFP plasmid used to produce the rAAV2/1-hVMD2-GFP and rAAV2/2-hVMD2-GFP vector constructs. (B). Map of the pTR-VMD2-BEST1 plasmid used to produce the rAAV2/1-hVMD2-cBEST1, rAAV2/2-hVMD2-cBEST1, rAAV2/1-hVMD2-hBEST1 and rAAV2/2-hVMD2-hBEST1 vector constructs. TR: AAV2 inverted terminal repeats; VMD2: human VMD2 promoter [32]; SV40 SD/SA: SV40 late viral protein gene 16S/19S splice donor and acceptor signal; hGFP: “humanized” green fluorescence protein reporter31; BEST1: coding sequence of wild-type canine BEST1 or wild-type human BEST1 gene; SV40 (poly A) and bGH poly (A): polyadenylation signals; HSK-tk: thymidine kinase promoter of the herpes simplex virus; Neo R: coding sequence of the neomycin resistance gene. (TIF) [file pone.0075666.s001.tif]

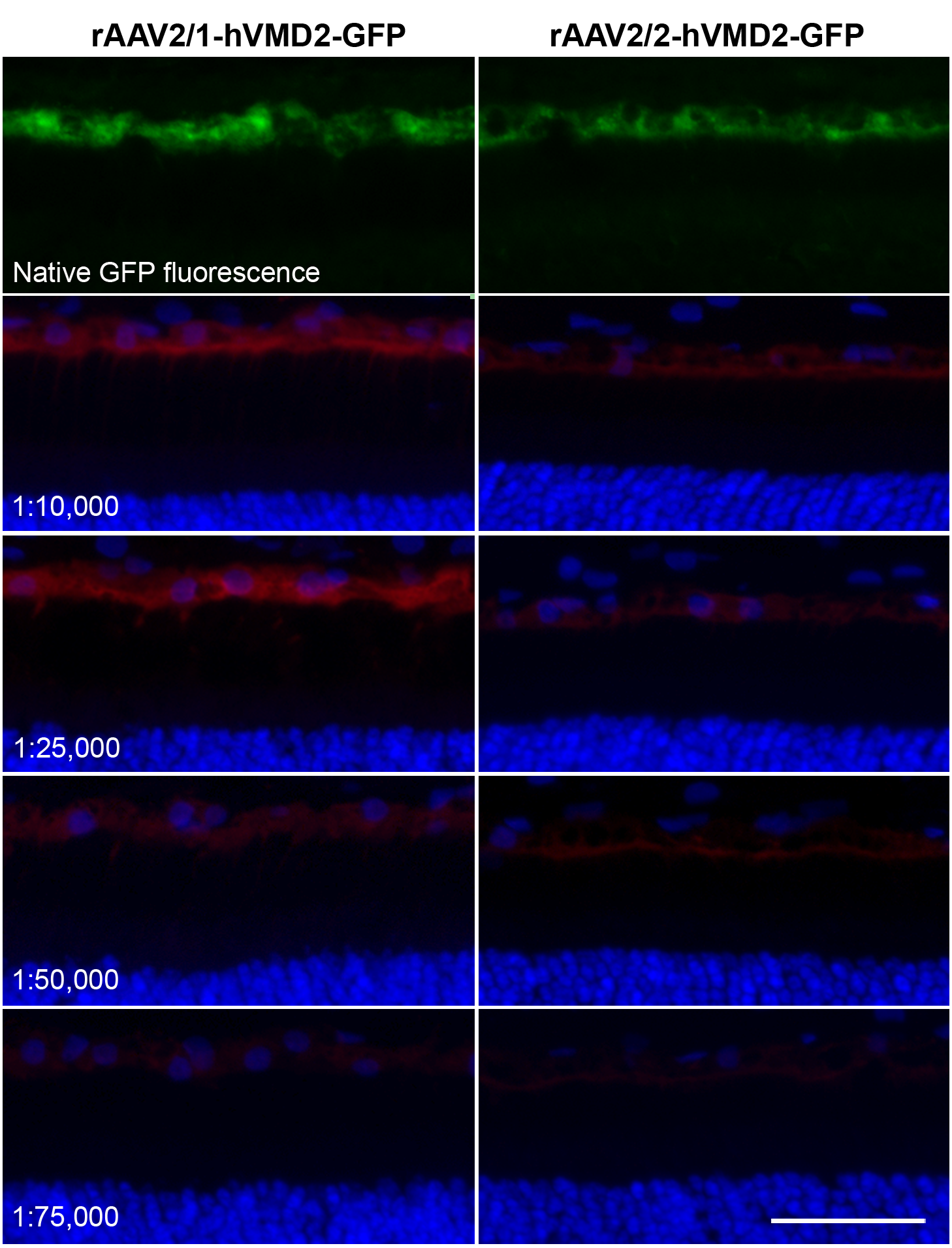

Supplement: Figure S2 — Transduction efficiency of rAAV2/1 and rAAV2/2 vectors carrying GFP reporter under control of human VMD2 promoter. Comparison of GFP expression levels induced by rAAV2/1 (2.63×10^11^ vg) or rAAV2/2 (9.11×10^10^ vg) at 6 weeks p.i. Native GFP expression (green) appeared more pronounced in the rAAV2/1 transduced RPE cells as confirmed by a dilution series of anti-GFP antibody (red). Expression levels induced with rAAV2/1 were detectable up to 1∶75,000 dilution (left panel), while the rAAV2/2-mediated expression could not be visualized beyond a 1∶50,000 dilution (right panel). Considering the difference in total virus genomes injected, however, both vectors appear to be qualitatively similar in transduction efficiency and both specifically target transgene expression to the RPE cell monolayer. Cell nuclei were stained with DAPI; vg: vector genomes injected; p.i.: post injection; scale bar: 40 µm. (TIF) [file pone.0075666.s002.tif]

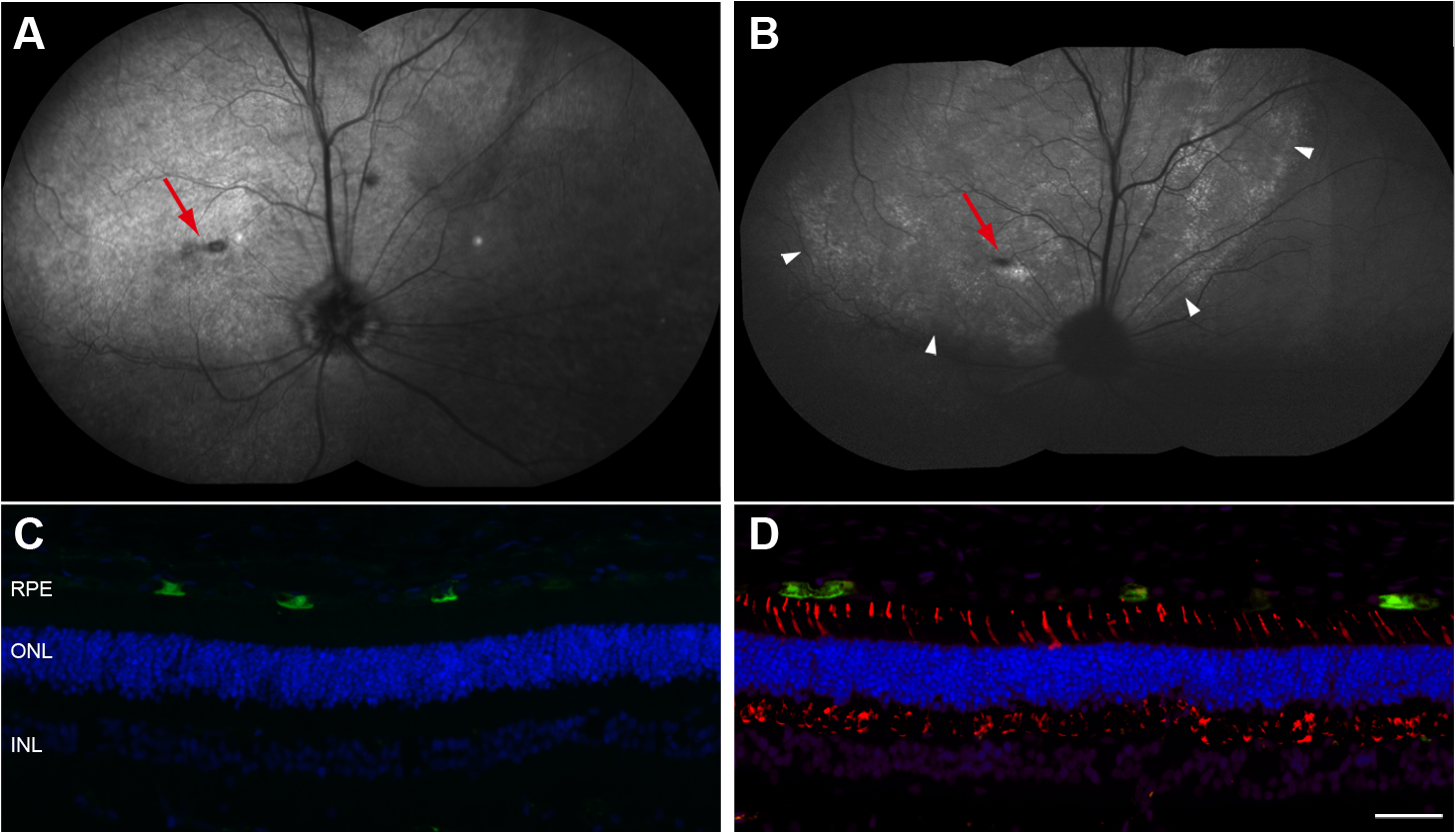

Supplement: Figure S3 — Monitoring the bleb kinetics and spatial extent of single subretinal injection in the canine fundus. (A–D) rAAV2/1-hVMD2-cBEST1-injected eye (1.94×10^11^ vg/ml) with a spike-in of corresponding vector expressing GFP (3.81×10^9^ vg/ml); a higher magnification figure of part of the fundus is shown in Fig. 4A. Composite fundus images NIR 55° 82°×56° (A) and AF 55° ART 98°x58° (B) captured 4 weeks after single subretinal injection of 150 µl. Note the GFP spiked area visible in autofluorescence mode (B) delimiting the spatial extent of injection (arrowheads). The arrow indicates retinotomy site. (C–D) Single (C) and double (D) immunolabeling of anti-GFP (green) and anti-hCAR (red). The GFP-positive cells scattered in the RPE monolayer corresponded to the injection boundaries outlined by AF mode (B). This area shows minimal damage to cones. Cell nuclei were stained with DAPI; scale bar: 40 µm. (TIF) [file pone.0075666.s003.tif]
