# Supplementary material for: Recombinant AAV-Mediated BEST1 Transfer to the Retinal Pigment Epithelium: Analysis of Serotype-Dependent Retinal Effects
Source: PLoS One. 2013 Oct 15;8(10):e75666. doi: 10.1371/journal.pone.0075666 (PMC3797066; doi:10.1371/journal.pone.0075666)
Supplement: Table S1 — Summary of injected subjects used in the studies. Recombinant adeno-associated virus vectors and dosage analyzed in total of 34 canine eyes. Eyes #: number of eyes injected per each type of vector; vg: vector genomes injected; Evaluation time point: post injection time by endpoint evaluation. p.i.: post injection. (DOCX) [file pone.0075666.s004.docx]

Table S1. Summary of injected subjects used in the studies.

| **Vector construct** | **Serotype** | **Promoter** | **Transgene** | **Titer vg/ml** | **Eyes #** | **Volume** | **vg injected** | **Evaluation time point** |
| --- | --- | --- | --- | --- | --- | --- | --- | --- |
| rAAV2-hVMD2-GFP | 1 | hVMD2 | GFP | 1.17x10^12^ - | 1 | 120µl | 2.64x10^11^ | 2 weeks p.i. |
|  |  |  |  | - 2.20x10^13^ | 1 | 150µl | 3.30x10^11^ | 2 weeks p.i. |
|  |  |  |  |  | 1 | 160µl | 2.80x10^11^ | 2 weeks p.i. |
|  |  |  |  |  | 1 | 120µl | 1.40x10^11^ | 4 weeks p.i. |
|  |  |  |  |  | 1 | 180µl | 1.58x10^11^ | 4 weeks p.i. |
|  |  |  |  |  | 1 | 150µl | 2.63x10^11^ | 4 weeks p.i. |
|  |  |  |  |  | 1 | 185µl | 3.35x10^12^ | 4 weeks p.i. |
|  |  |  |  |  | 1 | 190µl | 3.44x10^12^ | 4 weeks p.i. |
|  |  |  |  |  | 1 | 120µl | 1.40x10^11^ | 6 weeks p.i. |
|  |  |  |  |  | 1 | 150µl | 1.76x10^11^ | 6 weeks p.i. |
|  |  |  |  |  | 1 | 150µl | 2.63x10^11^ | 6 weeks p.i. |
|  |  |  |  |  | 1 | 150µl | 1.31x10^11^ | 6 weeks p.i. |
| rAAV2-hVMD2-GFP | 2 | hVMD2 | GFP | 6.07x10^11^ | 1 | 150µl | 9.11x10^10^ | 2 weeks p.i. |
|  |  |  |  |  | 1 | 150µl | 9.11x10^10^ | 2 weeks p.i. |
|  |  |  |  |  | 1 | 200µl | 1.21x10^11^ | 4 weeks p.i. |
|  |  |  |  |  | 1 | 150µl | 9.11x10^10^ | 6 weeks p.i. |
|  |  |  |  |  | 1 | 150µl | 9.11x10^10^ | 6 weeks p.i. |
|  |  |  |  |  | 1 | 150µl | 9.11x10^10^ | 6 months p.i. |

Table S1 Continued

| **Vector construct** | **Serotype** | **Promoter** | **Transgene** | **Titer vg/ml** | **Eyes #** | **Volume** | **vg injected** | **GFP component** | **Evaluation** |
| --- | --- | --- | --- | --- | --- | --- | --- | --- | --- |
| rAAV2-hVMD2-*BEST1* | 1 | hVMD2 | c*BEST1* | 1.29x10^12^ - | 1 | 120µl | 2.63x10^11^ | 1.74 x10^9^ - | 4 weeks p.i. |
|  |  |  |  | - 1.11x10^13^ | 1 | 140µl | 1.53x10^11^ | - 3.81x10^9^ | 4 weeks p.i. |
|  |  |  |  |  | 1 | 170µl | 2.19x10^11^ |  | 4 weeks p.i. |
|  |  |  |  |  | 1 | 180µl | 2.00x10^12^ |  | 4 weeks p.i. |
|  |  |  |  |  | 1 | 180µl | 2.00x10^12^ |  | 4 weeks p.i. |
|  |  |  |  |  | 1 | 150µl | 1.94x10^11^ |  | 6 weeks p.i. |
|  |  |  |  |  | 1* | 90µl | 1.92x10^11^ |  | 4 weeks p.i. |
|  |  |  |  |  | 1* | 100µl | 7.10x10^10^ |  | 4 weeks p.i. |
|  |  |  |  |  | 1* | 100µl | 1.07x10^11^ |  | 4 weeks p.i. |
| rAAV2-hVMD2-*BEST1* | 1 | hVMD2 | h*BEST1* | 6.85x10^12^ | 1 | 170µl | 1.16x10^12^ | 1.04x10^9^ | 4 weeks p.i. |
|  |  |  |  |  | 1* | 150µl | 1.03x10^12^ |  | 6 weeks p.i. |
| rAAV2-hVMD2-*BEST1* | 2 | hVMD2 | c*BEST1* | 2.61x10^12^ | 1 | 170µl | 3.92x10^11^ |  | 6 weeks p.i. |
|  |  |  |  |  | 1 | 150µl | 3.92x10^11^ |  | 6 months p.i. |
|  |  |  |  |  | 1* | 170µl | 4.44x10^11^ |  | 4 weeks p.i. |
| rAAV2-hVMD2-*BEST1* | 2 | hVMD2 | h*BEST1* | 5.88x10^12^ | 1 | 150µl | 8.82x10^11^ |  | 4 weeks p.i. |
|  |  |  |  |  | 1 | 170µl | 1.00x10^12^ |  | 6 weeks p.i. |

(*) represents *cmr1* carrier eyes; p.i. - post injection
