# Supplementary material for: Recombinant AAV-Mediated BEST1 Transfer to the Retinal Pigment Epithelium: Analysis of Serotype-Dependent Retinal Effects
Source: PLoS One. 2013 Oct 15;8(10):e75666. doi: 10.1371/journal.pone.0075666 (PMC3797066; doi:10.1371/journal.pone.0075666)
Supplement: Table S2 — List of primary antibodies used for immunolabeling. Tissue sections were washed in 1XPBS/0.25% TX-100 for 5 minutes and blocked for 1 h (10% normal goat serum, 1XPBS/0.25% TX-100, 0.05% sodium azide). Overnight incubation (at 4°C) with antibodies listed in the table was followed by three 1XPBS washes and incubation with 1∶200 Alexa Fluor 568 nm goat anti-rabbit (A11036, Invitrogen, Carlsbad, CA, USA) or goat anti-mouse (A11031, Invitrogen, Carlsbad, CA, USA) secondary antibody, respectively, for 1 h at room temperature. (DOC) [file pone.0075666.s005.doc]

Table S2. List of primary antibodies used for immunolabeling.

| **Antibody** | **Dilution** | **Source** |
| --- | --- | --- |
| Rabbit polyclonal anti-GFP | 1:1000-1:75000 | Courtesy of W.C. Smith (University of Florida) |
| Mouse monoclonal anti-GFP | 1:1000 | MAB3580, Chemicon, Billerica, MA, USA |
| Mouse monoclonal anti-Best1 | 1:400 | Ab2182, Abcam, Cambridge, MA, USA |
| Mouse monoclonal anti-RPE65 | 1:500 | NB100-355, Novus Biologicals, LLC, Littleton, CO, USA |
| Mouse monoclonal anti-Rho | 1:1000 | MAB5316, Millipore, Billerica, MA, USA |
| Rabbit polyclonal anti-hCAR | 1:10000 | Courtesy of C.M. Craft (University of Southern California) |
| Rabbit polyclonal anti-red/green opsin | 1:100 | AB5405, Millipore, Billerica, MA, USA |
| Rabbit polyclonal anti-blue opsin | 1:5000 | AB5407, Millipore, Billerica, MA, USA |
